# Neonatal diabetes mellitus and congenital diaphragmatic hernia: coincidence or concurrent etiology?

**DOI:** 10.1186/1687-9856-2012-21

**Published:** 2012-07-10

**Authors:** Emmanuelle S Topiol, Laurie A Minarich, Charles A Williams, Roberto T Zori, David W Kays, Michael J Haller

**Affiliations:** 1 University of Florida, Department of Pediatrics, Division of Endocrinology, PO Box 100296, Gainesville, FL, 32610, USA; 2 University of Florida, Department of Pediatrics, Division of Genetics, PO Box 100296, Gainesville, FL, 32610, USA; 3 University of Florida, Department of Surgery, Division of Pediatric Surgery, PO Box 100119, Gainesville, FL, 32610, USA

**Keywords:** Neonatal diabetes mellitus, Congenital diaphragmatic hernia, PLAGL1, Imprinting, Duplication

## Abstract

Neonatal diabetes mellitus (NDM) is a rare metabolic disorder, affecting approximately 1 in 500,000 live births. The management of NDM is challenging, as the benefits of controlling hyperglycemia must be balanced with the risks of iatrogenic hypoglycemia. NDM occurs in both permanent and transient forms, which have been genetically and phenotypically well characterized. Herein, we present the previously unreported combination of transient NDM (TNDM) and congenital diaphragmatic hernia (CDH). In addition to reviewing the management and genetics of NDM we discuss the potential for overlapping genetic or embryologic abnormalities to explain the concurrence of CDH and NDM.

## Background

Neonatal diabetes mellitus (NDM) is a rare disorder of glucose metabolism (affecting 1 in 500,000 live births) and may be either transient or permanent [1]. Affected neonates frequently present with hyperglycemia, intrauterine growth retardation, and variable degrees of dehydration. Despite the relative severity of insulin deficiency, ketoacidosis is uncommon. Treatment with exogenous insulin is required to promote normal growth and avoid acute and sub-acute complications associated with severe hyperglycemia.

Transient NDM (TNDM) is associated with over-expression of paternal genes on chromosome 6 that reduce the capacity of the β cell to release insulin. TNDM is characterized by resolution of hyperglycemia by 18 months of age though 40-50% of patients with TNDM experience a recurrence of diabetes in adolescence or early adulthood [1,2]. Permanent NDM (PNDM) is associated with mutations of the β cell ATP-sensitive potassium channel which disable the β cell’s ability to depolarize and release insulin [3].

Congenital diaphragmatic hernia (CDH) occurs in approximately 1 in 2,500 children and results from a developmental defect in the diaphragm that allows abdominal viscera to penetrate the chest [4]. Because the herniating spleen, liver, and intestines may compress lung tissue during critical periods of lung organogenesis, CHD results in varying degrees of irreversible pulmonary hypoplasia. Approximately 50% of CDH cases are associated with chromosomal abnormalities or congenital malformations including gut malrotation, umbilical hernia, hydronephrosis, cardiac defects, and type 1 diabetes [2,4,5]. However, the combination of CDH and TNDM has *not* previously been reported.

Given the unlikely coincidence of CDH and TNDM we explore the potential for a common genetic or embryologic etiology of these two diagnoses.

## Case presentation

A 2.4 kg female infant was born at 38 weeks gestation to a 28-year-old primigravida via elective cesarean section. Birth weight was 2401 grams (2nd percentile), length was 48 cm (29th percentile), and head circumference was 34 cm (32nd percentile). The baby was diagnosed with a left sided CDH at 14 weeks gestation by routine prenatal ultrasound. Due to the prenatally diagnosed CDH, the baby was intubated at birth, made nothing per os, and placed on intravenous hyperalimentation providing a glucose infusion rate (GIR) of 8 mg/kg/min. Although her blood glucose concentration was normal at birth (103 mg/dl), she developed marked hyperglycemia (493 mg/dl) at 9 hours of life.

Family history was notable for neonatal diabetes in the paternal grandmother that resolved within 2 months. She developed adult-onset diabetes in the 4^th^ decade of life despite a lean body habitus.

Pertinent physical findings in our patient included shallow supraorbital ridges, relatively prominent eyes, a mildly protruding tongue with micrognathia, mild pectus excavatum, and bilateral accessory nipples. She had a normal cranial shape, no central forehead nevus flammeus, normal ears without pits, and no evidence of limb asymmetry. She had normally developed female genitalia and an otherwise normal skin exam (See Additional file 1).

Initial laboratory evaluation revealed negative urine ketones, undetectable serum insulin concentration (< 0.5 mcIU/ml), and a low serum C-peptide concentration (0.2 ng/mL). Arterial blood gas and electrolyte measurements demonstrated a non-anion gap metabolic acidosis with an elevated serum lactate (2.47 mmol/L). A thoracoabdominal ultrasound performed on day of life 2 revealed a pancreas without definite abnormality, but bowel gas prevented optimal delineation of the organ.

Initial management included decreasing the GIR, but a concomitant decrease in blood glucose was not observed. Over the next 6 hours, she was treated with 3 subcutaneous injections of 0.5 units rapid-acting insulin analog, which failed to normalize her blood glucose concentration. A continuous intravenous infusion of regular insulin was started with an initial rate 0.04 units/kg/hour. Six hours after initiating the insulin infusion, her blood glucose concentration fell below 200 mg/dl and insulin was briefly discontinued. Her blood glucose concentration gradually increased to 230 mg/dl over the next 6 hours, and a simultaneous serum insulin level was <0.5 mcIU/ml. Therapy with continuous intravenous insulin was resumed at 0.03 units/kg/hour. The rate of the infusion was then titrated to achieve blood glucose concentrations between 200–300 mg/dl. The maximum rate of insulin administration was 0.12 units/kg/hour.

On day of life 4, blood glucose concentrations were stable on 0.02-0.05 units of insulin per kg per hour and our patient underwent surgical repair of her CDH. At repair she had a moderately severe hernia with small bowel, colon, stomach, and spleen in the left chest. Her CDH was associated with a 50% loss of normal diaphragm and was repaired with a patch. Her left lung was approximately 30% of normal size. She transitioned to oral feeds approximately one week after the operation.

In preparation for discharge home, our patient was transitioned to continuous subcutaneous insulin infusion (SCII) therapy with rapid-acting analog diluted 10-fold. A single basal rate of 0.5 units/hr of U-10 insulin was programmed in the pump (total daily dose 1.2 units). Blood glucose was monitored every 3 hours (typically before breastfeeding) and a correction bolus of insulin was given for blood glucose values greater than 350 mg/dL. Use of a continuous glucose monitoring system was considered, but our patient had so little subcutaneous fat that sensor placement was deemed impossible. Upon discharge, her insulin requirement quickly waned, and the insulin was discontinued at 7 weeks of age.

At her three-month visit, she was thriving, with a weight of 4004 grams, and a hemoglobin A1c of 4.7%. Comparative genomic hybridization microarray analysis using a 180 K oligonucleotide array platform revealed a 389 K micro-duplication in region 6q24.2, which includes the paternally expressed PLAGL1 (pleomorphic adenoma of the salivary gland gene like 1) and HYMAI (hydatidiform mole associated and imprinted [non-protein coding]) genes known to be associated with TNDM. An additional gene, PHACTR2 (phosphatase and actin regulator 2), also maps within the same deletion boundary, though presumably it is not related to the problems in our patient. The same duplication was found in her father, who did not have neonatal diabetes and who does not have considerable hyperglycemia, as evidenced by a normal HbA1c (personal communication, MJ Haller).

The same micro-duplication in region 6q24.2 was discovered in our patient’s paternal uncle and his unborn child. Amniocentesis demonstrated the presence of the mutation in our patient’s first cousin. At birth, the baby boy followed a very similar course to our patient. He was small at birth, weighing 1710 grams at 34 weeks gestation (5^th^ percentile). Hyperglycemia developed in the first 24 hours of life, and insulin therapy was required for 6 weeks before resolution occurred.

## Discussion

The management of infants with NDM remains challenging, given the need to balance the benefits of normal glycemia with the risks of recurrent hypoglycemia in infants. In our experience, continuous subcutaneous insulin infusion (CSII) therapy provides optimal equipoise in managing children with NDM. While there are limited outcomes data to drive evidence based treatment recommendations for NDM, several case series have been published. Tubina-Rufi et al. reported on their experiences in managing NDM over 18 years, and found CSII therapy to be most effective in safely managing diabetes in newborns requiring insulin for more than 15 days [6].

TNDM is most commonly associated with over-expression of the paternally inherited PLAGL1 and HYMAI genes at the 6q24 locus. PLAGL1 is a zinc finger DNA binding protein with tumor suppressor activity, and overexpression of this gene has been shown to arrest β cell division and induce apoptosis. HYMAI shares a promoter region with PLAGL1, and methylation of this promoter on the maternal allele results in sole expression of the paternally inherited allele in most tissues; HYMAI is a non-coding RNA gene whose function is unknown. Over-expression of these genes can occur as a result of uniparental disomy of chromosome 6, duplication of 6q24 of the paternal allele, or hypomethylation of the maternal PLAGL1/HYMAI differentially methylated region [7,8].

PNDM is most commonly caused by mutations in the *KCNJ11* and *ABCC8* genes, which encode the Kir6.2, and SUR1 subunits of the β cell ATP-sensitive potassium channel, respectively [3]. This potassium channel is constitutively open, but closes in response to increased intracellular ATP levels that occur as a consequence of hyperglycemia. When the channel closes, β cell depolarization occurs, and insulin is released. A gain-of-function mutation in this channel prevents closure, so that the β cell remains hyperpolarized and unable to secrete insulin. Evaluation of the specific mutations resulting in PNDM is imperative, as patients with KCNJ11 mutations can be safely and effectively managed with oral sulfonylurea therapy [9]. Failure to appropriately diagnose these patients may result in unnecessary lifelong therapy with subcutaneous insulin.

Emerging knowledge of the mutations underlying PNDM and TNDM has improved our capacity to provide genetic counseling to affected families. While most mutations causing PLAGL1 overexpression are due to de novo events such as methylation defects or segmental uniparental disomy, microduplications can be inherited in an autosomal dominant, imprinting-type, manner [7,10,11]. In families with this imprinting type of NDM, like our case, overexpression of PLAGL1 only occurs when the microduplication is passed through the male germ line, since passage through the maternal germ line will inactivate both copies of PLAGL1 via methylation of its promoter [12,13]. Dominant imprinting inheritance was supported in our family by the observation that the patient’s father and his brother (the paternal uncle) both carried the duplication but were asymptomatic. However, in the grandparental generation, the paternal grandmother had neonatal DM, presumably indicating that she carried the duplication.

Despite improved understanding of the genetic basis for NDM, diagnosis of NDM has historically been a post-partum event based on the unexpected development of persistent hyperglycemia in a neonate. Genetic testing is typically performed well after initiation of insulin, with results often reported after discharge from the nursery. The potential to diagnose these mutations prenatally, as occurred with our patient’s cousin, may greatly improve the management of TNDM by allowing families and physicians to be optimally prepared for the development of hyperglycemia. Only recently has uniparental disomy of paternal chromosome 6 been detected through amniocentesis [14].

Finally, the combination of CDH and TNDM has *not* previously been reported. A review of diaphragmatic hernia genomic etiologies did not implicate the 6q24.2 locus [15]. However, CDH has been reported in association with numerous extra-diaphragmatic abnormalities including gut malrotation, umbilical hernia, hydronephrosis, cardiac defects, and type 1 diabetes. CDH is also a common feature of Beckwith-Wiedemann syndrome (BWS), another imprinted genetic disorder that, like TND, may be associated with macroglossia and umbilical hernia [16]. The known association of CDH and extra-diaphragmatic abnormalities suggests that a common genetic or embryologic etiology might underlie CDH and TNDM.

## Conclusions

This case provided the opportunity to demonstrate the successful use of CSII in the management of TNDM complicated by a concurrent congenital anomaly. In addition, this case models the potential value of genetic counseling and prenatal diagnosis in families affected by NDM. Given the incidence of TNDM and CDH, the likelihood of a child presenting with both CDH and TNDM is approximately 1 in 1,250,000,000. While a review a review of diaphragmatic hernia genomic etiologies did not implicate the 6q24.2 locus, our case either represents the observation of an extremely rare coincidental event or indicates that there is a potential link between TNDM and CDH. Further examination of loci neighboring the 6q24 region, and their role in organogenesis, may provide insights toward explaining our patient’s development of two uncommon diagnoses.

## Consent

Written informed consent was obtained from the patient for publication of this Case report and any accompanying images. A copy of the written consent is available for review by the Editor-in-Chief of this journal.

## Abbreviations

BWS, Beckwith-Wiedemann syndrome; CDH, Congenital diaphragmatic hernia; CSII, Continuous subcutaneous insulin infusion; HYMAI, Hydatidiform mole associated and imprinted [non-protein coding]) genes; NDM, Neonatal diabetes mellitus; PHACTR2, Phosphatase and actin regulator 2; PLAGL1, Pleomorphic adenoma of the salivary gland gene like 1; PNDM, Permanent neonatal diabetes mellitus; TNDM, Transient neonatal diabetes mellitus.

## Competing interests

The authors declare that they have no competing interests.

## Authors’ contributions

MJH, LM, CAW, and DAK managed the patient during hospitalization. EST, LM, and MJH drafted the manuscript. CAW and RTZ edited the manuscript and contributed to the discussion. All authors read and approved the final manuscript.

## Supplementary Material

Additional file 1 Pertinent physical findings in our patient included shallow supraorbital ridges, relatively prominent eyes, a mildly protruding tongue with micrognathia, mild pectus excavatum, and bilateral accessory nipples. She had a normal cranial shape, no central forehead nevus flammeus, normal ears without pits, and no evidence of limb asymmetry. Click here for file
