# Supplementary figures and images for: Neonatal diabetes mellitus and congenital diaphragmatic hernia: coincidence or concurrent etiology?
Source: Int J Pediatr Endocrinol. 2012 Jul 10;2012(1):21. doi: 10.1186/1687-9856-2012-21 (PMC3408326; doi:10.1186/1687-9856-2012-21)

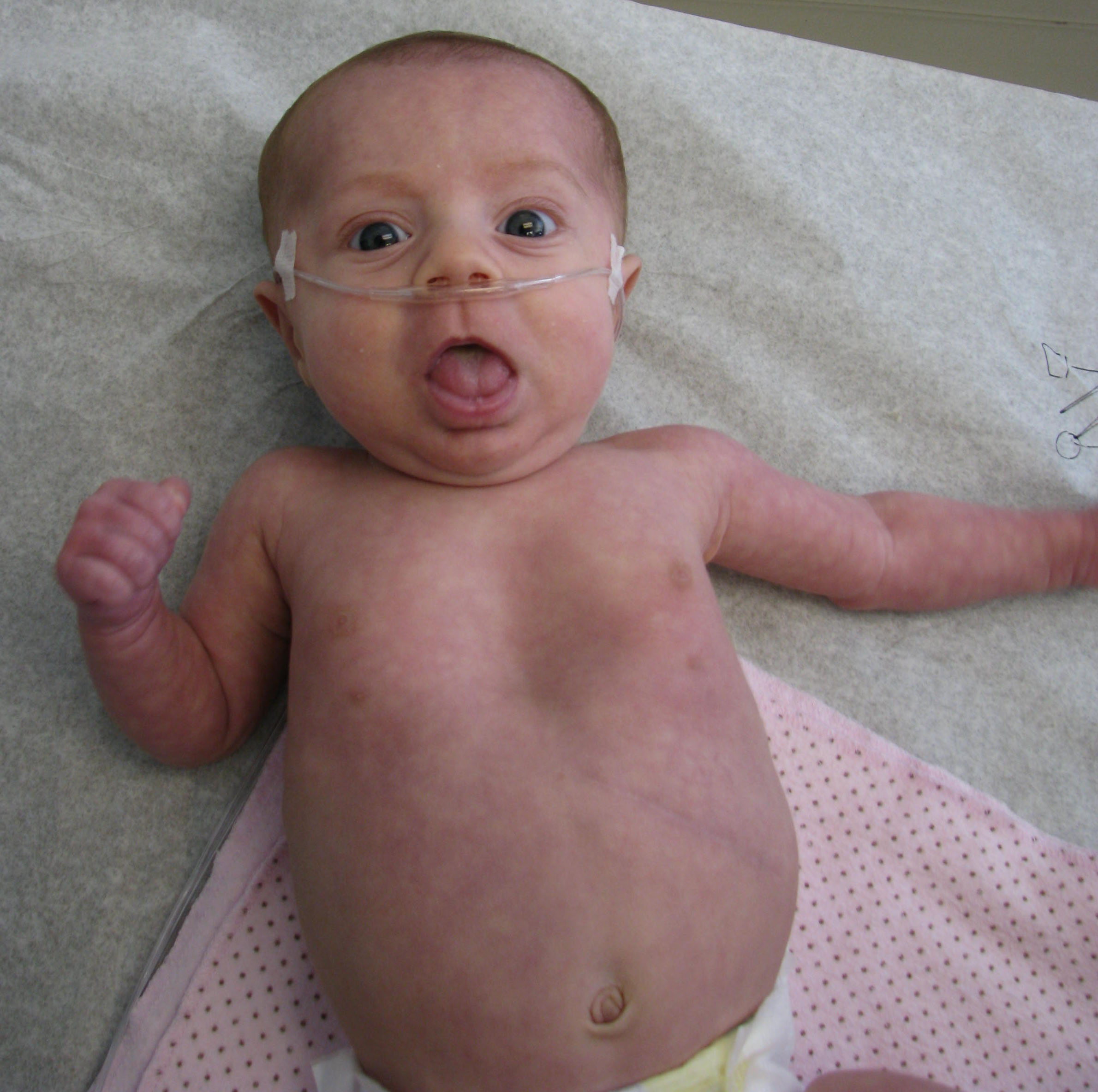

Supplement: Additional file 1 — Pertinent physical findings in our patient included shallow supraorbital ridges, relatively prominent eyes, a mildly protruding tongue with micrognathia, mild pectus excavatum, and bilateral accessory nipples. She had a normal cranial shape, no central forehead nevus flammeus, normal ears without pits, and no evidence of limb asymmetry. [file 1687-9856-2012-21-S1.jpeg]
